# Strengthening the Substrates of Wood Single Lap Joints Using a Novel Hot-Melt Film Adhesive to Mitigate Delamination

**DOI:** 10.3390/ma19081547

**Published:** 2026-04-13

**Authors:** Francisco C. C. Ribeiro, Shahin Jalali, Vasco C. M. B. Rodrigues, Ricardo J. C. Carbas, Eduardo A. S. Marques, Fengzhen Sun, Lucas F. M. da Silva

**Affiliations:** 1 Department of Mechanical Engineering, Faculty of Engineering, University of Porto, 4200-465 Porto, Portugal; 2 Institute of Science and Innovation in Mechanical and Industrial Engineering (INEGI), Faculty of Engineering, University of Porto, 4200-465 Porto, Portugal; 3 School of Mechanical Engineering, Tongji University, Shanghai 201804, China

**Keywords:** strain-rate sensitivity, impact performance, delamination, adhesive bonding, hybrid wood substrates, numerical analysis

## Abstract

Delamination remains a critical limitation in the structural application of wood, particularly in adhesively bonded joints. This study investigates the use of a cyclic olefin-based hot-melt film adhesive (Zeon^®^ LS-XU) as a thermoplastic interlayer as a means to delay delamination and enhance joint performance. Single lap joints (SLJs) were tested under quasi-static (1 mm/min) and impact (3 m/s) loading to assess strain-rate effects. Six configurations were examined: two reference, two toughened (with an additional 15 mm of adhesive on each overlap side) and two hybrid configurations combining oak (*Quercus alba*) and pine (*Pinus pinaster* Aiton) substrates to improve stress wave propagation. A finite element elastic model was developed to analyse stress distributions and explain the superior performance of hybrid joints. Results revealed that the thermoplastic interlayer delayed delamination onset and increased energy absorption, while hybrid configurations achieved more uniform stress distributions and significantly higher strengths under dynamic loading. The most effective configuration, the hybrid joint under impact conditions, represents a strength improvement of approximately 84% of the peak load compared to the pine reference joints. Overall, introducing a thermoplastic interlayer offers an efficient and lightweight strategy to enhance the toughness and reliability of wood joints exposed to variable loading conditions.

## 1. Introduction

Adhesive bonding technology is gaining prominence as a compelling alternative to traditional mechanical joining methods across numerous industries, including automotive, aerospace and electronics. Compared to conventional joining techniques such as riveting or fastening, adhesive bonding offers notable advantages: it enables a continuous bond line, facilitating more uniform stress distribution and enhancing both load transfer and fatigue resistance. These features contribute to reduced overall weight and cost. Moreover, adhesive bonding allows for the joining of dissimilar materials and offers greater flexibility in joint design [1,2,3,4].

Wood, a naturally anisotropic material, presents an environmentally sustainable option for a variety of structural applications, even in domains where materials like steel, aluminum or concrete have long been dominant. Its renewed appeal stems from its low carbon footprint and renewable nature, particularly when sourced through responsible forestry practices that safeguard long-term biodiversity and forest health [5,6]. Among the most attractive candidates for engineering applications are fast-growing wood species, valued for their rapid renewability. However, these woods often present limited mechanical performance, making them less suitable for demanding applications without further enhancement [3,7,8].

Wood structures are particularly sensitive to discontinuities such as holes, notches or penetrations created by nails and screws. These discontinuities introduce local stress concentrations that cut through the fibres, disrupting the natural load-carrying capacity of the cellular structure and often leading to premature or even catastrophic failure. Moreover, the geometry of wood and its fibre orientation must be carefully considered, since mechanical behaviour is strongly direction dependent [9]. Recent studies highlight that introducing defects or altering the substrate microstructure accelerates delamination and weakens adhesive joints, further underlining the importance of avoiding stress concentrators in load-bearing wooden assemblies [10,11]. It is important to distinguish delamination in wood from interlaminar delamination in fibre-reinforced composite laminates. In composites, delamination arises from interlaminar tensile and shear stresses between fibre plies of differing stiffness or orientation. In wood, however, delamination is governed primarily by the inherently weak transverse tensile and shear strength of the cellular microstructure along growth-ring boundaries and is strongly influenced by moisture gradients, grain angle variations and local density differences. Unlike composites, wood does not possess a well-defined interlaminar fracture toughness in the classical sense: instead, crack propagation follows the path of least resistance through the early wood zones, making failure highly sensitive to environmental and processing conditions.

Wood joints play a pivotal role in structural integrity and are commonly achieved through adhesive bonding [12]. A key metric in assessing joint quality is shear strength, which is influenced by the adhesive’s chemical and physical properties and the wood’s microstructure, as well as its chemical, physical and mechanical characteristics and the process parameters [13,14]. The wood failure percentage (WFP) is another widely used indicator; it is a figure obtained after the shear test that aims to determine the strength of the glue bond. Higher WFP values generally reflect stronger joints, where failure occurs predominantly in the wood itself rather than at the adhesive interface [15,16].

In previous studies, strategies such as wood densification via thermo-hydromechanical (THM) treatments have been used to improve mechanical properties and bonding performance [17,18,19]. However, such processes are often associated with drawbacks like moisture sensitivity and set-recovery, which can lead to dimensional instability under cyclic environmental conditions [18,20]. While these methods offer material enhancement, they also introduce complexity and limitations in real-world applications [20].

In contrast, the present study explores an alternative approach that avoids the use of densified wood. Instead, different configurations of natural wood substrates with the use of a new hot-melt adhesive (HMA) are proposed and investigated with the goal of minimizing delamination in adhesively bonded joints. This approach addresses one of the key failure modes in wood bonding, delamination, by examining how layer arrangement influences joint performance. One effective strategy for enhancing the peel strength of single lap joints (SLJs) is to employ a matrix material with high transverse toughness, which improves resistance to peel-induced stresses. This method can be further supported by design modifications aimed at reducing localized stress concentrations in the composite adherends. Ramezani et al. [21] examined the mechanical performance of hybrid composite joints reinforced with different laminate configurations. Their review highlighted several approaches to strengthening SLJs, including the use of tougher matrices and transversely toughened systems. In their study, composite adherends were paired with various laminate materials such as aluminum, polymers and thin-ply composites. Results demonstrated that thin-ply laminates enhanced joint strength by approximately 10% and helped alleviate peel stress near the overlap ends. The researchers concluded that hybrid joints combining different laminate types can deliver advantages in terms of weight reduction, damage tolerance and load transfer efficiency. Shang et al. [15] developed a method to mitigate delamination problems in adhesive joints using composite substrates. Their research combined experimental tests and numerical analyses to evaluate joint strength, failure mechanisms and stress distribution in SLJs. The incorporation of a toughened composite material led to a notable 22% improvement in joint strength and altered the dominant failure mode from adherend delamination to cohesive failure within the adhesive layer. The findings suggest that employing toughened composites in adhesive joints effectively reduces delamination risks and enhances the overall structural performance.

The novelty of this work lies in combining a cyclic olefin-based hot-melt adhesive film with hybrid pine-oak layups. This approach offers a lightweight alternative to wood densification by using an HMA and alternating pine-oak layers, avoiding issues such as set-recovery, dimensional instability and high processing requirements. It also enables a simpler manufacturing process that preserves the natural wood structure while enhancing joint performance. Single lap joints were selected due to their industrial relevance and ease of standardization. The study integrates experimental testing with finite element analysis to evaluate joint behaviour and stress concentrations, providing a critical assessment of the thermoplastic adhesive film for improving structural reliability and reducing delamination in composite wood assemblies.

## 2. Experimental Details

To systematically evaluate the performance of the proposed reinforcement strategies, a comprehensive experimental procedure was designed. This included the preparation of single lap joints using pine and oak substrates, bonded with a thermoplastic hot-melt adhesive film, under different joint configurations. Mechanical testing was performed under both quasi-static and impact loading conditions to capture the strain-rate sensitivity of the adhesive-wood interface. The following section details the materials, manufacturing procedures and testing protocols employed.

### 2.1. Adherends

#### 2.1.1. Pine Wood

The primary material under investigation was pine timber (*Pinus pinaster* Aiton), sourced from the southern region of Alentejo, Portugal, consistent with the provenance used in Oliveira and Moura [22]. Timber samples included both sapwood and heartwood and were harvested from mature trees aged approximately 40 to 50 years. The material was obtained from the trunk at breast height (1.3 m above ground) to represent typical structural timber properties.

The beams used to produce the adherends for the single lap joints had a cross-section of 100 × 25 mm, although the thickness varied when manufacturing the hybrid specimens (1 and 4 mm). It should be noted that achieving perfect symmetry of the growth rings relative to a central vertical axis was not always feasible. The air-dry density of *Pinus pinaster* Aiton used in this study was 540 ± 40 kg/m^3^ and the equilibrium moisture content at standard laboratory conditions (20 °C, 50% RH) was about 12% and 18% as supplied. A total of three pine trunks were used to produce the timber beams from which all pine adherend specimens were cut.

Oliveira and Moura [22] have previously published the values of the nine elastic constants and strength properties on the longitudinal (L), radial (R) and tangential (T) directions for pine wood. These properties are summarized in Table 1.

The notation adopted in the table is defined as longitudinal Young’s modulus (E_L_), radial Young’s modulus (E_R_), transverse Young’s modulus (E_T_), Poisson’s ratio (ν), shear modulus in the longitudinal-radial plane (*G_LR_*), shear modulus in the longitudinal-transverse plane (*G_LT_*), shear modulus in the transverse-radial plane (*G_TR_*), maximum longitudinal strength (*σ_L_*), maximum radial strength (*σ_R_*) and maximum transverse strength (*σ_T_*).

#### 2.1.2. Oak Wood

The second material utilized during this research was oak wood (*Quercus alba*), which was one of the materials used for the SLJ substrates. The oak wood was sourced from a certified commercial Portuguese timber supplier, from the central-northern region of Portugal, obtained from two trunks within a single batch to minimize between-batch variability. The cross-section dimensions of the oak wood beams were the same as the pine wood ones. The elastic properties of oak wood were obtained from Oliveira and Moura [22] and Reiterer et al. [23], as presented in Table 2. The moisture content ranged from 8% to 25%, with all substrates between 12% and 18% as supplied. Dry wood (≤19% moisture) ensures stability, strength and biological resistance. Wood specimens had a density of 500 ± 30 kg/m^3^ during assembly.

### 2.2. Adhesive

The 0.4 mm thick cyclic olefin-based thermoplastic adhesive film named LS-XU is multi-material adhesive (MMA) developed by Zeon^®^ Corporation (Chiyoda-ku, Tokyo, Japan) and was used in this study. It is a hot-melt film designed for multi-material bonding applications.

For quasi-static conditions, the mechanical properties of the Zeon adhesive have been characterized in parallel to this work. A summary of the mechanical properties relevant to the adhesive performance under quasi-static conditions, as well as the remaining shear properties required for the numerical simulation of the single lap joint, is provided in Table 3.

The symbols in the table are defined as yield strength (*σ_y_*), ultimate tensile strength (*σ_f_*), strain at failure (*ε*), shear strength (*τ*) and shear strain at failure (*γ*).

### 2.3. Single Lap Joint: Geometry and Manufacture

In order to manufacture the specimens, the adhesive was processed according to the manufacturer’s guidelines, curing at 150 °C at 10 bar for 1 h, followed by a gradual cooling at ambient temperature with the press and heating system being turned off.

Three distinct configurations of wooden SLJs were designed and manufactured for this study.

**Figure 1:**
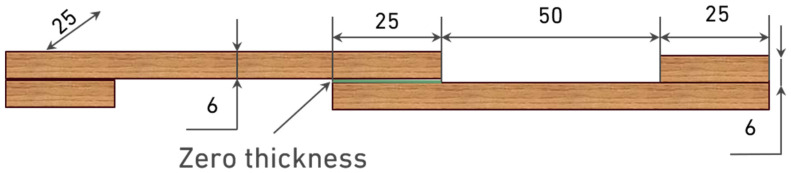
Geometry of the reference SLJ configuration (dimensions in mm).

**Reference configuration**: The first configuration, referred to as the reference joint, was fabricated using either pine or oak substrates with a uniform thickness of 6 mm. The geometry of this configuration is presented in Figure 1.

**Figure 2:**
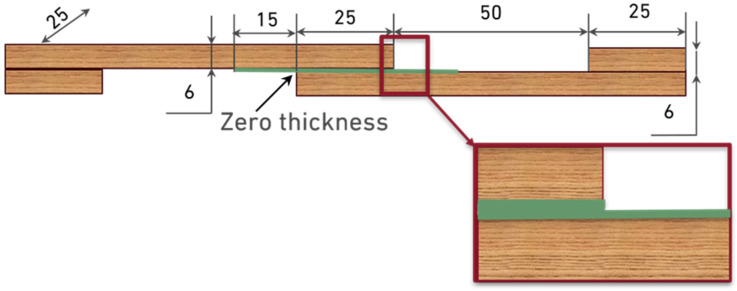
Geometry of the toughened SLJ configuration (dimensions in mm).

**Toughened configuration**: The second configuration, also prepared with both pine and oak adherends of 6 mm thickness, incorporated an additional adhesive layer extending 15 mm beyond each end of the overlap (Figure 2). This design aimed to take advantage of adhesive penetration into the porous wood structure. Such penetration increases the effective bonding area and locally reinforces the wood cell walls, which improves transverse strength in the overlap region. As a result, stress concentrations at the overlap ends can be reduced, delaying crack initiation, limiting propagation along the bondline and ultimately enhancing the joint’s capacity for energy absorption and toughness.

**Figure 3:**
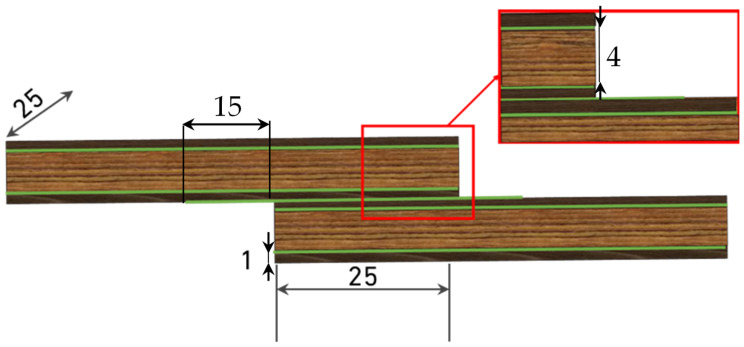
Geometry of the hybrid SLJ configurations (dimensions in mm).

**Hybrid configuration**: The third configuration combined pine and oak within the same adherend, producing hybrid specimens (Figure 3). Two thickness arrangements were manufactured: (i) oak in the core (4 mm) with pine plies on the outer surfaces (1 mm each), forming an O-P-O configuration, and (ii) pine in the core (4 mm) with oak plies on the outer surfaces (1 mm each), forming a P-O-P configuration. Adhesive layers were applied between the different wood plies, with penetration further strengthening the interlaminar regions. The hybrid design was intended to improve stress distribution along the overlap length and increase the overall load-bearing efficiency of the joints.

The manufacturing process for the SLJs bonded with HMA film followed a controlled procedure. An iron mould coated with release agent was employed to facilitate removal of the specimens after curing. The wood plies were positioned in the mould between alignment pins to ensure accurate specimen width. For the hybrid configurations, the stacking sequence alternated between wood layers and adhesive film, following the designated material arrangement (O-P-O or P-O-P). Spacers were placed at the overlap region to guarantee a uniform overlap length across all specimens.

The adhesive was applied at nominally zero thickness, a condition that promotes penetration into the wood structure and minimizes the risk of insufficient adhesion. To achieve uniform consolidation, a hot press was used to apply constant pressure over the entire bonding area. Six SLJs were manufactured simultaneously under the same cycle.

### 2.4. Testing Conditions

In this study, five specimens were tested for each loading condition and configuration. Quasi-static SLJ tests were carried out on an 8801 servo-hydraulic testing machine (Instron, Norwood, MA, USA) equipped with a 100 kN load cell at a constant displacement rate of 1 mm/min in accordance with ASTM D5868-01 [24].

Impact tests were conducted on a custom-built drop-weight apparatus [25] capable of releasing a 56 kg mass from a height of up to 1.27 m (equivalent to an impact velocity of 5 m/s). For the present work, a 10 kg mass was dropped at 3 m/s, corresponding to an impact energy of 45 J, in order to be able to compare with previous results where the same drop-weight energy was used.

Displacement was obtained by trapezoidal integration of the load–time data and validated through high-speed video recordings coupled with Digital Image Correlation (DIC). To enable accurate tracking, specimens were first coated with a white paint base layer and subsequently speckled with black paint, allowing the high-speed camera to capture the movement of the speckle pattern across successive frames and determine the corresponding displacements.

## 3. Numerical Models

Alongside the experimental campaign, numerical simulations were developed to provide deeper insights into stress distributions in the different joint configurations. The numerical models were developed in ABAQUS^®^ software (2017 version), with the finite element (FE) analysis carried out to understand the improvements made when using the hybrid joints under impact conditions. Two distinct models were created: one for quasi-static conditions and one for impact. The primary objective of the simulation was to evaluate the effect of substrate hybridization and adhesive toughening on the stress distribution along the bondline, rather than to model progressive failure or delamination mechanisms. The FE model was therefore limited to purely elastic material behaviour, serving as a qualitative tool to identify regions of stress concentration that could contribute to delayed delamination observed experimentally. A preliminary mesh refinement check was conducted to confirm that the predicted stress distributions and load–displacement responses were stable with respect to element size. The numerical model was therefore used primarily as a comparative and interpretive tool to assess how hybridization and adhesive modification influence stress redistribution within the joint. Future studies will focus on detailed mesh convergence and experimental strain validation to further substantiate the modelling results.

**Figure 4:**
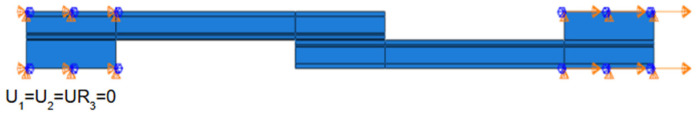
Modelled boundary conditions.

Both models were constructed as 2D planar deformable shell parts, and the boundary conditions are illustrated in Figure 4. At one extremity, an encastre was applied to replicate the gripping system. For the opposite end of the SLJ, in the case of quasi-static loading (tensile testing), a displacement of 1 mm was imposed, and for impact conditions, a mass (drop-weight) equivalent to the experimental impactor was modelled and assigned the same initial velocity of 3 m/s. This velocity field was defined in the initial step and propagated throughout the subsequent analysis.

For the quasi-static conditions, a static general analysis was used, when it came to the step type, while for the impact condition, a dynamic explicit step was employed in the simulation.

The material properties reported in the experimental details section were used as input for the numerical model. For the simulations, only one reference configuration (oak) and one hybrid configuration (P-O-P) were analysed to ensure that the substrate core remained consistent throughout the study.

No toughened configurations were taken into account in this study, because the exact properties of the toughened wood within the adhesive penetration zone, as well as the penetration depth itself, were unknown; therefore, it was not feasible to obtain reliable results for this case.

Selecting the optimal reinforcement strategy to mitigate delamination in wood joints is a complex task. In this study, different configurations were examined, namely reference joints and hybrid joints combining oak and pine layers. The primary goal was to redistribute stresses away from critical regions at the ends of the overlap, where delamination is most likely to initiate and to spread them more evenly across the adhesive–wood interface and throughout the core ply of the substrate.

Since there is additional adhesive introduced between the different wood layers in the hybrid configurations, bonding relies solely on the resin of the hot-melt film and delamination may still occur at the wood plies. The substrate configuration, therefore, plays a crucial role in reducing stress concentrations. By placing stiffer oak plies in the core (P-O-P), for example, the weak region is shifted away from the overlap ends into the core of the substrate, under impact conditions, lowering the magnitude of peel stresses. At the same time, the stiffer inner layers minimize transverse deformation and transmit lower peel stresses due to the adhesive-wood transition.

While material selection can strongly influence this process, this study focused on pine and oak as representative species. Consequently, the design of the joints required a compromise between maintaining lightweight, renewable substrates and achieving sufficient stiffness to prevent premature delamination at the wood layers.

Finally, the mechanical properties of the adhesive film in the overlap are also critical to the behaviour of SLJs and to the stress distribution across the transverse direction. To address this multi-variable problem and to better understand the influence of the substrate configuration, numerical models were developed under fully elastic assumptions, without cohesive damage laws. By applying a displacement-controlled boundary condition equivalent to the experimental loading, the peel stresses along the adhesive layer were extracted using the path tool. Following the same method, the shear stress along the substrate thickness was also extracted, utilizing once again the path tool.

### Numerical Results

**Figure 5:**
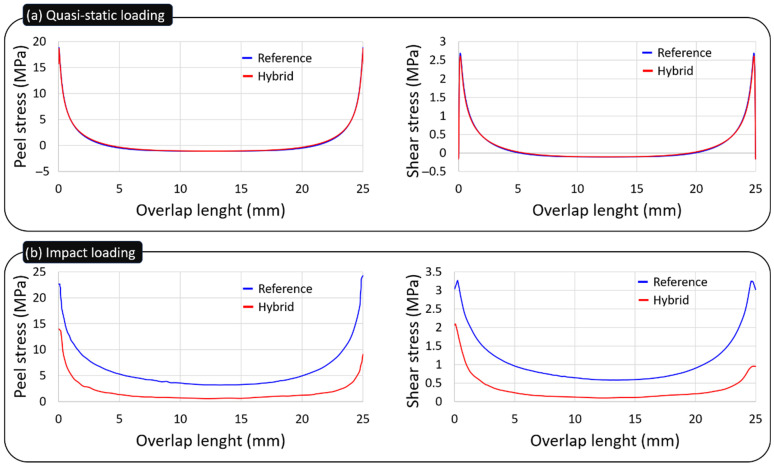
Peel and shear stresses along the adhesive layer under: (**a**) quasi-static loading; (**b**) impact loading.

The results in Figure 5a show that, at the same displacement under quasi-static loading, the maximum peel stress in the adhesive layer is essentially unchanged between the reference oak joint and the P-O-P hybrid (15.75 MPa versus 15.80 MPa). This indicates that, without stress-wave effects, which are present under impact conditions, the hybrid layup has limited influence on the substrate response.

Regarding the results under impact conditions, as Figure 5b demonstrates, the hybrid configurations show much lower peak peel stresses when compared with the reference configuration. The maximum peel stress obtained with the hybrid joint simulation was 13.95 MPa, and for the reference simulation it was 22.60 Mpa, showing a 38.27% decrease.

Similarly to the peel stress analysis, investigating the shear stress along the adhesive layer, for the same imposed displacement under quasi-static loading, Figure 5a demonstrates that the peak shear stress in the adhesive layer remains virtually the same for the reference oak joint and the P-O-P hybrid, 2.69 MPa and 2.61 MPa, respectively. This suggests that, in the absence of the stress-wave phenomena associated with impact, the hybrid stacking sequence has only a modest effect on the joint’s response. By contrast, the impact results in Figure 5b show that the hybrid configuration develops markedly lower peak shear stresses than the reference joint. In the simulations, the hybrid joint reached a maximum shear stress of 3.26 MPa, while the reference attained 2.10 MPa, amounting to a 35.58% reduction when it comes to the peak shear stress at the adhesive layer.

Since the peel stresses at the adhesive layer are a major driver of delamination, it is possible to conclude that, even though delamination was not prevented, there was a delay on the delamination and propagation of the stress in the hybrid joint, in consideration of the 38.27% decrease in the maximum peel stress from the reference to the hybrid configuration. Therefore, hybrid configurations can delay delamination by transmitting lower peel stresses, which reduces substrate rotation and bending and explains the improved impact performance.

**Figure 6:**
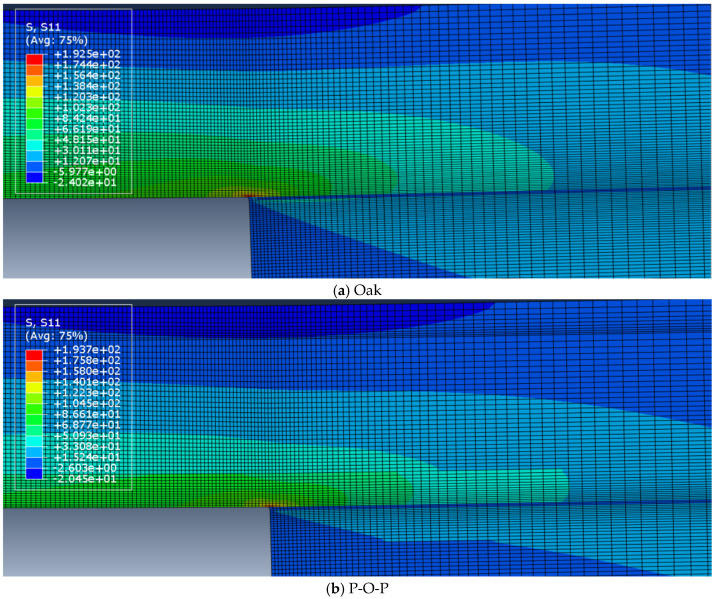
Tensile stress distribution at the substrate. Oak (**a**) and P-O-P (**b**).

Furthermore, Figure 6 shows a comparison of the tensile stress distribution, at quasi-static test conditions, between the reference (oak: at the top) and the hybrid (P-O-P: at the bottom) and configurations under numerical study.

Analysing the tensile stress through the thickness of the substrate (Figure 6) under static conditions, it is noticeable that there are no significant improvements when using the hybrid configuration because the values of the stress are similar in both cases. However, it is also important to note the discontinuities of the stress distribution in the hybrid joint due to the interface between pine and oak wood.

**Figure 7:**
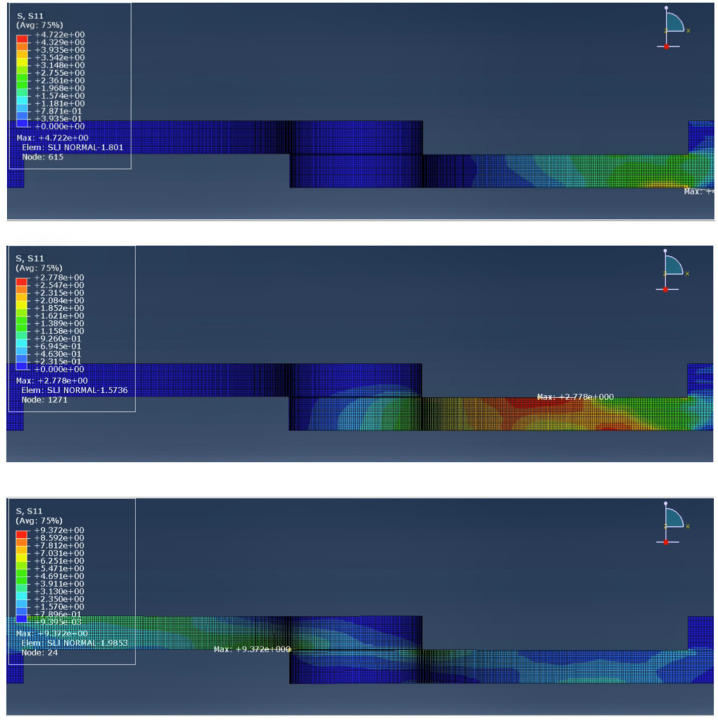
Sequence showing the stress wave propagation throughout the reference joint.

**Figure 8:**
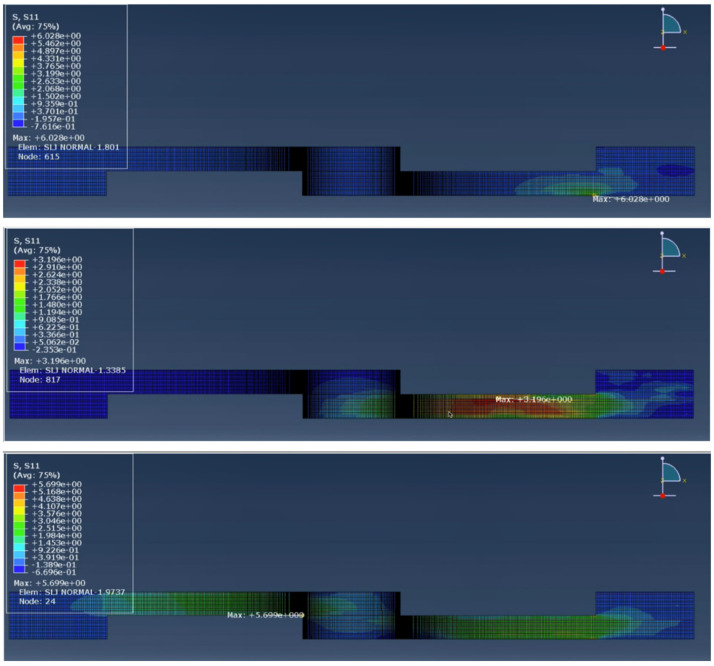
Sequence showing the stress wave propagation throughout the hybrid joint.

In the impact testing of the hybrid configurations, a significant improvement in joint performance was observed compared to the reference specimens. Figure 7 and Figure 8 demonstrate the stress wave propagation, over time, throughout the whole joint, for the reference and hybrid configurations, respectively.

When analysing the stress wave propagation along the length of the joints for both modelled configurations, even though the maximum stress is roughly the same, it is notable that in the hybrid configuration, the stress propagates along the inner layer of the substrate, along the core, while in the reference model, the stress wave propagates along the edges of the wood. This will uniformize the energy absorption, concentrate the stress in stronger areas and allow the substrate to deform more evenly before failure.

Overall, as previously reported for the stresses at the adhesive layer, the numerical results, regarding the stress distribution, show no improvement of the hybrid joint when compared with the reference joint under quasi-static testing. Contrarily, when the joints are submitted to impact loading, the use of a hybrid configuration with both types of wood highlights its ability to better distribute the stresses along the joint length, showing great improvement when compared to the reference configuration.

## 4. Experimental Results and Discussion

This section presents the experimental findings obtained from the reference, toughened and hybrid joint configurations under both quasi-static and impact loading conditions. The results are analysed in terms of load-displacement response, failure load and failure modes, enabling a direct comparison of the influence of substrate configuration and reinforcement strategy on joint performance, experimentally and numerically.

### 4.1. Reference Joints

**Figure 9:**
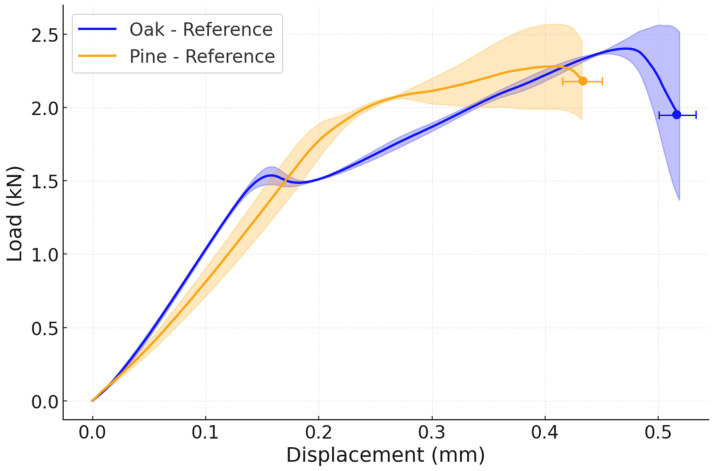
Experimental P-δ curves under quasi-static conditions for both reference configurations:oak and pine (the highlighted area corresponds to the standard deviation).

An average representative load–displacement curve from the quasi-static experiments for the reference configurations, using pine and oak wood species. The nominal shear strength, calculated as maximum load divided by the overlap area (625 mm^2^), was 3.87 ± 0.11 MPa for the reference oak joint and 3.65 ± 0.50 MPa for the reference pine joint under quasi-static loading. These values are consistent with shear strengths reported in the literature for adhesively bonded wood joints of similar geometry, typically in the range of 3 to 5 MPa [6,15]. Failure under quasi-static conditions was predominantly adhesive for all configurations (wood failure percentage, WFP ≈ 0%), indicating that the wood-adhesive interface governed the response, as shown in Figure 9. The failure load of both reference joints is very similar, as well as their displacement, since the stiffnesses of both woods are also comparable. Specifically, the average failure load of a reference oak joint is 2.41 ± 0.07 kN, while the average failure load of the reference pine joint is 2.28 ± 0.31 kN.

**Figure 10:**
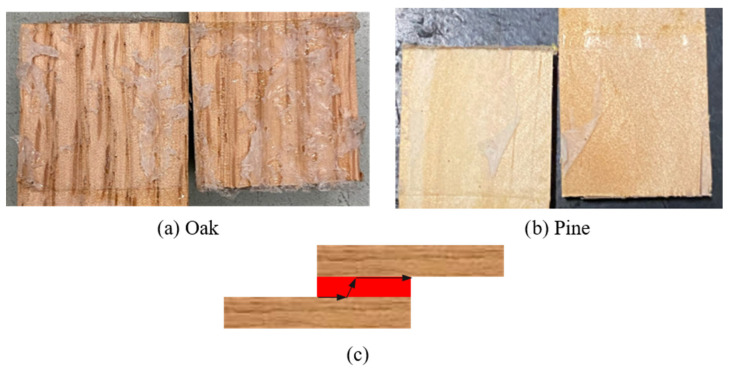
Failure modes obtained for reference joints under quasi-static conditions: (**a**) oak wood; (**b**) pine wood; (**c**) crack path scheme for both reference configurations.

Both wood reference joints failed adhesively, showing that the bond between the adhesive and the wood is weak. The failure modes of both joints are shown in Figure 10, together with a scheme illustrating the crack path that was obtained while testing the reference configurations under quasi-static loading (the red part represents the adhesive while the black arrows represent the crack path).

**Figure 11:**
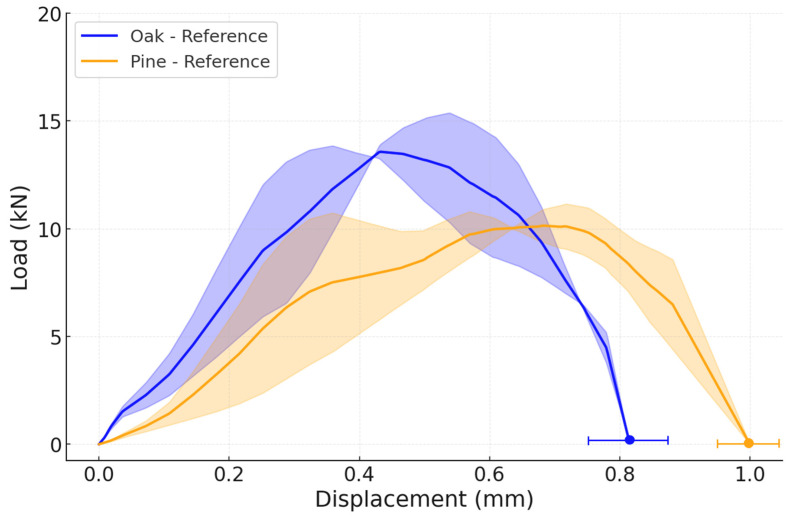
Experimental P-δ curves under impact conditions for both reference configurations: oak and pine (the highlighted area corresponds to the standard deviation).

**Figure 12:**
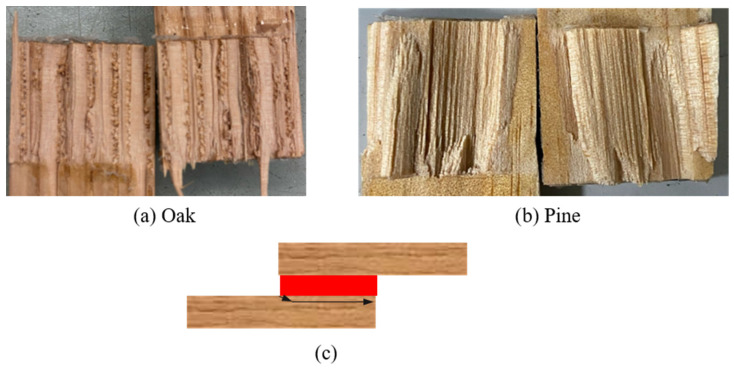
Failure modes obtained for reference joints under impact conditions: (**a**) oak wood; (**b**) pine wood; (**c**) crack path scheme for both reference configurations.

Regarding the impact performance of the reference joints, the average failure load was 13.58 ± 1.77 kN for the oak joints, just above the results obtained for the pine joints, which present a failure load of 10.23 ± 0.90 kN (Figure 11). Under impact conditions, both reference joints failed by delamination in the wood, shown in Figure 12 with a scheme of the crack path, estimating WFP ≈ 100% and confirming that the dynamic loading rate shifted the governing failure mechanism from interface-dominated to wood-dominated, consistent with the strain-rate sensitivity. Notably, the pine joint also exhibited a substantially greater displacement, which would result in a small increase in energy absorbed of approximately 16%.

Noticeably, the performance of both wood types while using the reference configurations is very similar under quasi-static conditions, since the failure mode is adhesive. When submitted to impact conditions, pine wood has a lower failure load, due to its lower strength, but it shows higher ductility when compared with oak wood.

### 4.2. Toughened Joints

Nevertheless, the average failure load of the oak wood slightly increased to 2.70 ± 0.21 kN, representing a growth of about 12 ± 9% relative to the reference joints. This increase is likely due to the effect of the extra adhesive that was added to the substrate. Regarding the pine wood, the average failure load of the toughened configuration decreased to 2.09 ± 0.07 kN, which represents a reduction of 8 ± 12% when compared to the reference specimens. This smaller reduction shows that the additional adhesive has a higher impact on the oak substrates, which is due to the fact that oak wood is more porous, and therefore, the adhesive can more easily penetrate the oak wood. The shear strength of the toughened oak joint under quasi-static loading was 4.32 ± 0.37 MPa, whereas for pine, 3.34 ± 0.11 MPa was reported. The marginal difference in quasi-static performance between toughened and reference configurations, despite the additional 15 mm of adhesive at the overlap edges, is consistent with the adhesive-governed failure mode (WFP ≈ 0%) observed for all quasi-static specimens. When adhesive failure governs, extending the adhesive coverage does not substantially increase the failure load because the limiting factor is the interfacial shear strength per unit area, not the total bonded length [14].

**Figure 13:**
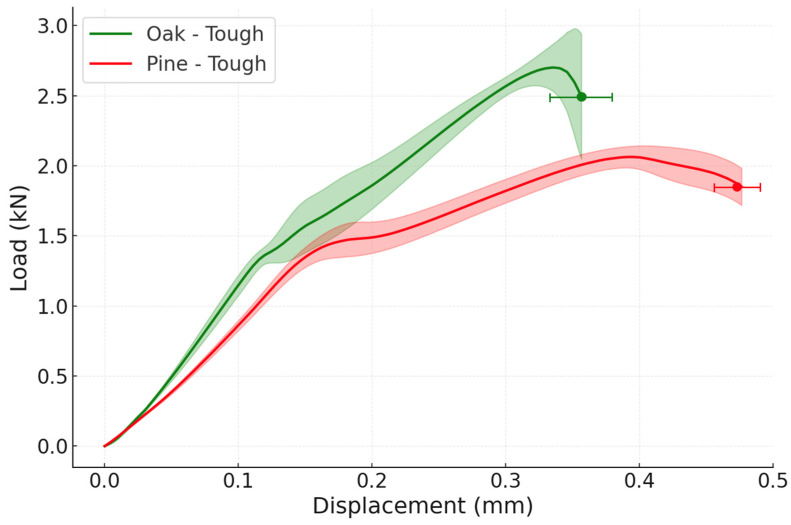
Experimental P-δ curves under quasi-static conditions for both reference and toughened configurations (the highlighted area corresponds to the standard deviation).

Finally, Figure 13 presents a comparison of the experimental load-displacement curves between both wood species (pine and oak), presenting the aforementioned results.

**Figure 14:**
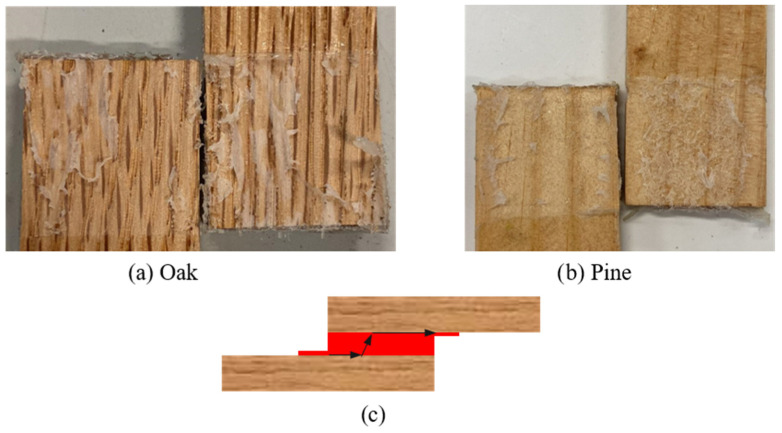
Failure modes obtained for toughened joints under quasi-static conditions: (**a**) oak wood; (**b**) pine wood; (**c**) crack path scheme for both toughened configurations.

As mentioned before, the failure mode for both toughened configurations was also adhesive failure (Figure 14), which once again shows that the interface is the weakest link in the joint. The crack path obtained for the toughened configurations under quasi-static testing conditions is also illustrated.

**Figure 15:**
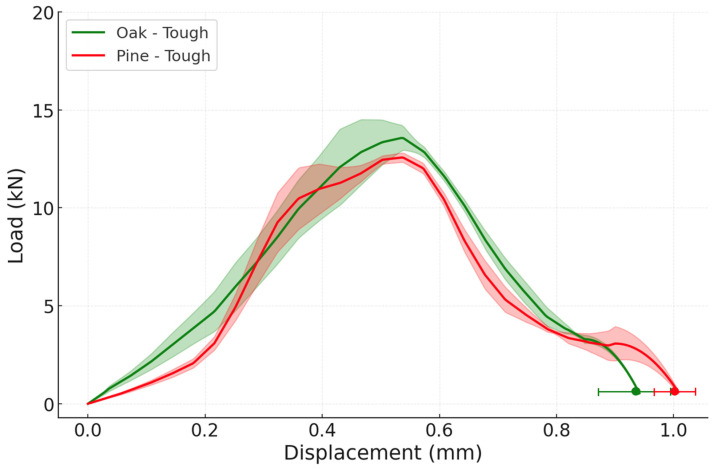
Experimental P-δ curves under impact conditions for both reference and toughened configurations (the highlighted area corresponds to the standard deviation).

When studying the impact performance of the toughened joints, a failure load of 13.59 ± 0.90 kN was obtained for the oak specimens, while for the pine specimens, a failure load of 12.57 ± 0.29 kN was acquired. This represents, on the one hand, practically no improvement in the oak wood joints, but, on the other hand, an increase of around 23 ± 11% in the pine wood joints when compared to the reference, as depicted in Figure 15.

**Figure 16:**
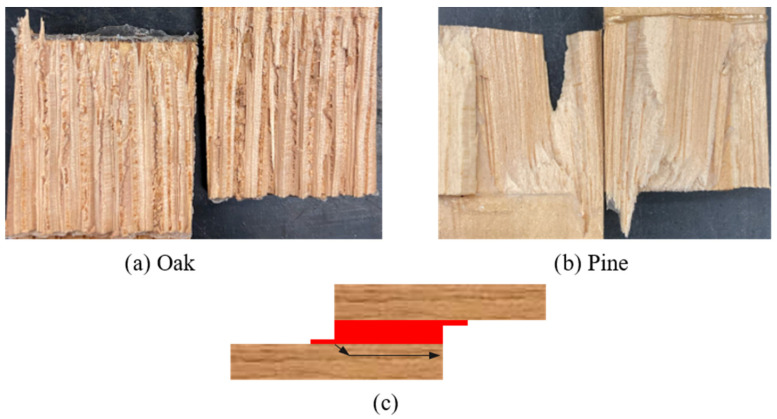
Failure modes obtained for reference joints under impact conditions: (**a**) oak wood; (**b**) pine wood; (**c**) crack path scheme for both toughened configurations.

Concerning the failure mode of the toughened joints under impact testing, it is visible that the failure was, once more, delamination in the wood (Figure 16). The crack path is illustrated in the same figure. The toughened pine joint exhibited a higher failure load than the reference pine, with WFP increasing to approximately 100%, indicating a greater degree of wood involvement, consistent with the toughened interlayer distributing peel stresses more effectively at higher strain rates.

Under quasi-static loading, both wood species exhibited comparable performance in the reference configurations, since failure occurred adhesively. In contrast, under impact conditions, pine wood, despite the addition of extra adhesive in the toughened configuration, still showed a lower failure load than both oak configurations, due to its lower intrinsic strength, while simultaneously demonstrating greater ductility than the oak wood joints.

### 4.3. Hybrid Joints

**Figure 17:**
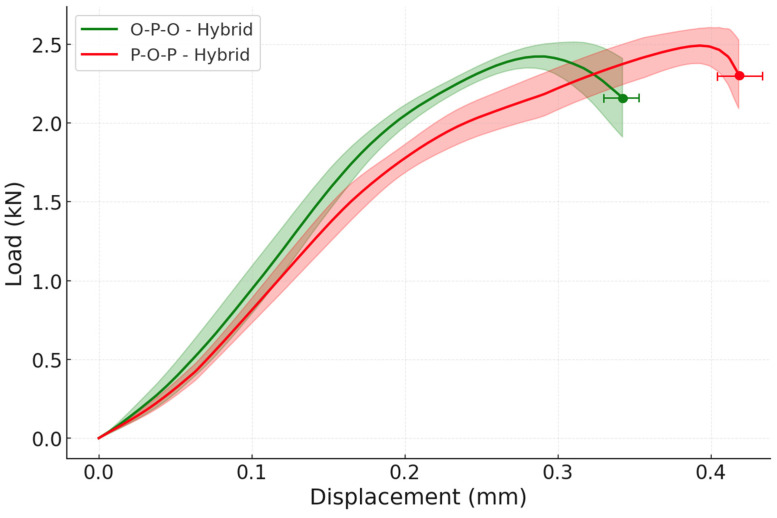
Experimental P-δ curves under quasi-static conditions for both reference and hybrid configurations (the highlighted area corresponds to the standard deviation).

Lastly, the hybrid configurations were evaluated. These joints under static conditions showed very small improvements when compared to the reference configurations and their load-displacement curves are shown in Figure 17.

The joints that used oak wood in the core of the substrate showed a slightly higher failure load, of 2.49 ± 0.12 kN (3.98 ± 0.19 MPa), when compared to the O-P-O hybrid configuration, which has a failure load of 2.43 ± 0.09 kN (3.89 ± 0.14 MPa). This can be explained by the fact that the oak wood is stronger than the pine wood and the P-O-P hybrid has a core layer composed of oak wood, making it slightly stronger than the O-P-O hybrid joint. These experimental results corroborate the numerical outcome, where the hybrid joints showed limited improvements at quasi-static conditions.

**Figure 18:**
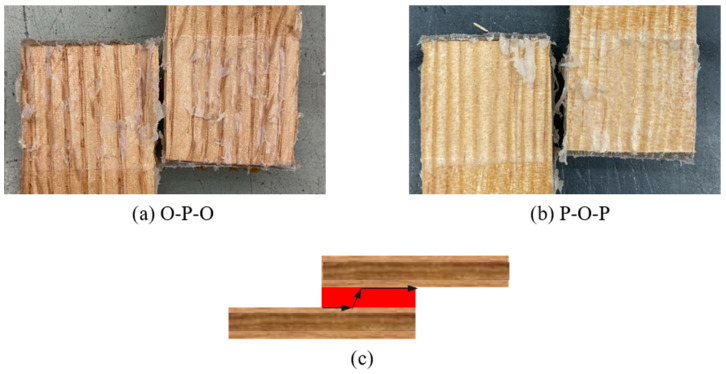
Failure modes obtained for hybrid joints under quasi-static conditions: (**a**) oak-pine-oak; (**b**) pine-oak-pine; (**c**) crack path scheme for both hybrid configurations.

It should also be noted that the failure mode for both hybrid configurations under quasi-static conditions was, once more, adhesive. The failure surface of the pair is presented in Figure 18, as well as the crack path for both joint configurations.

When it comes to the impact testing of the hybrid configurations, there is a considerable improvement in the joint behaviour when confronted with the reference specimens. This was to be expected due to the numerical analysis previously done, which showed major improvements for the hybrid configurations under impact loading.

**Figure 19:**
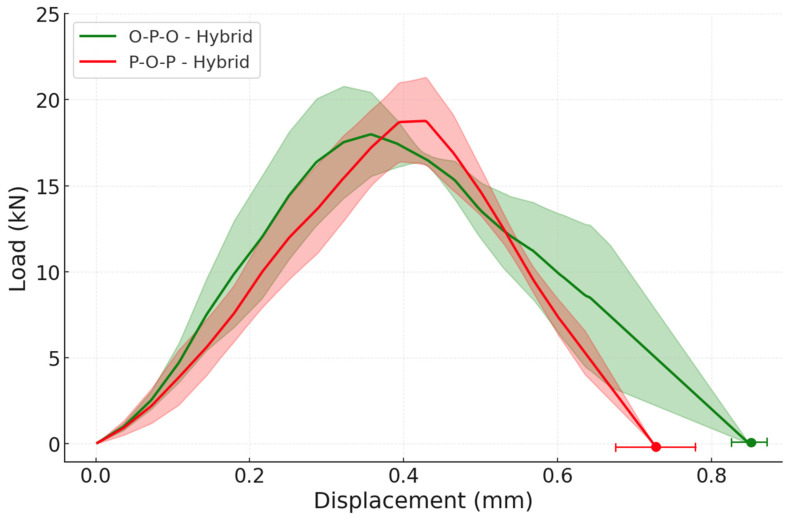
Experimental P-δ curves under impact conditions for both reference and hybrid configurations (the highlighted area corresponds to the standard deviation).

For instance, the O-P-O hybrid exhibited a failure load of 18.04 ± 1.81 kN and the P-O-P hybrid displays a failure load of 18.84 ± 2.32 kN; these results represent, for the O-P-O configuration, a 33 ± 22% increase when compared with the reference oak and a 76 ± 23% gain when compared with the reference pine and for the P-O-P configuration, a 39 ± 24% improvement when weighed against oak reference specimens and a 84 ± 28% increase when contrasted against the pine reference. The results are shown in the load-displacement graph of Figure 19. Under impact loading, all hybrid configurations failed by wood delamination (WFP ≈ 100%), confirming that the adhesive bond was stronger than the wood substrate under dynamic conditions.

**Figure 20:**
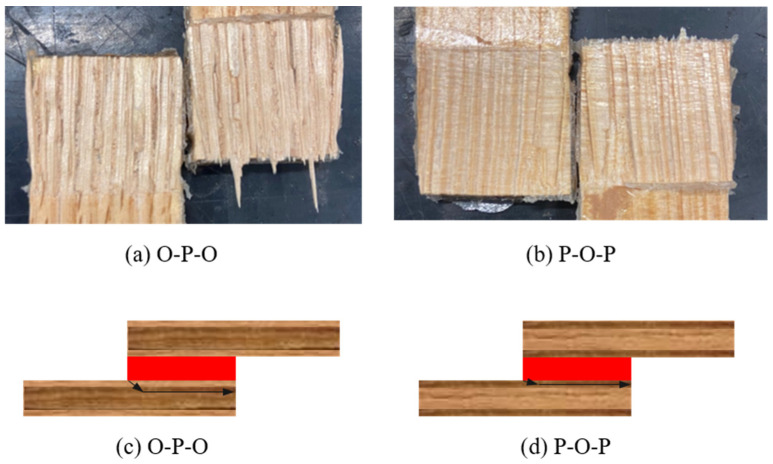
Failure modes obtained for hybrid joints under impact conditions: (**a**) oak–pine–oak; (**b**) pine–oak–pine; rack path scheme for both hybrid configurations (**c**) oak-pine-oak and (**d**) pine-oak-pine.

While there is a big improvement regarding the joint behaviour and specifically the failure load, the failure mode of the hybrid joints under impact was still delamination on the wood, as shown in Figure 20. It should be noted that in both hybrid configurations, the failure occurred in the pine layers of the substrate: this is due to the fact that pine wood is slightly weaker than the oak wood, making the joint fail in its layers.

Finally, although the hybrid configurations did not represent an upgrade when it comes to quasi-static conditions, when they were tested under impact conditions, the use of both types of wood and their ability to better distribute the stresses along the joint length proved to be very influential, showing great improvement in relation to the reference and also to the toughened. These experimental findings are consistent with the numerical simulations, which similarly highlighted the enhanced stress redistribution and delayed delamination in the hybrid layups, thereby validating the predictive capability of the developed models.

The high WFP under impact conditions indicates that the adhesive bond strength exceeded the wood substrate strength under dynamic conditions, a positive outcome confirming the effectiveness of the cyclic olefin HMA film in transferring load to the adherends. The absence of adhesive-line failure in any hybrid or reference joint under impact suggests that the HMA film formed a competent bond with both pine and oak substrates and that future performance improvements should focus on substrate modification rather than adhesive reformulation. This was also observed for the previously tested configurations.

**Figure 21:**
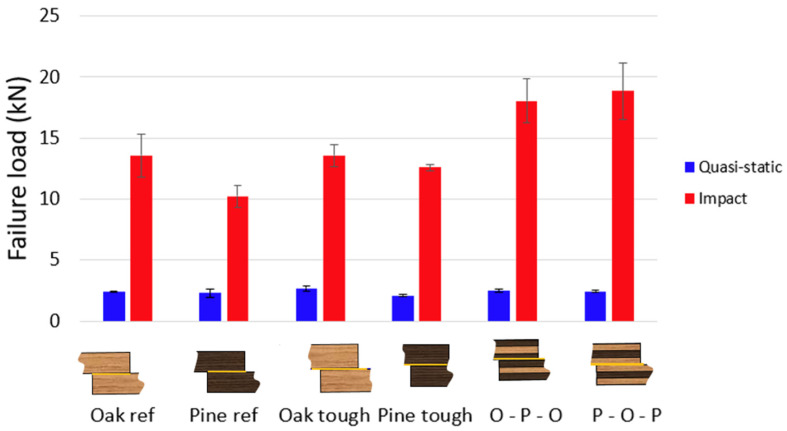
Summary chart of the experimental results.

In order to make a summary of the entirety of the experimental results obtained in this study, Figure 21 provides a concise overview of the quasi-static performance of all tested configurations with respect to failure load. It is worth noting that all joints tested under quasi-static loading failed adhesively, which helps explain the similarity of the results and the limited influence of the toughened and hybrid configurations, since the strength is mostly governed by the wood-adhesive interface rather than by the substrate reinforcement strategy.

Similarly, Figure 21 also summarizes the corresponding results under impact conditions. As expected, all configurations exhibited higher failure loads under impact compared to quasi-static loading, owing to the strain-rate sensitivity of the adhesives. Furthermore, it can be inferred that the hybrid configurations are much more strain-rate dependent, showing a greater improvement. It should also be emphasized that, under impact loading, all joints failed by delamination within the wood. In this case, the reinforcement strategies became more relevant, with the hybrid configuration providing the most significant benefits, while the toughened configuration showed only marginal improvements.

Ultimately, these experimental results are further supported by the numerical analyses previously performed, which likewise demonstrated improved joint behaviour in the hybrid configurations under impact loading, while not showing any improvement under quasi-static testing conditions, reinforcing the reliability of the predictive models developed.

## 5. Conclusions

This work evaluated whether a cyclic olefin-based hot-melt adhesive film (Zeon^®^ LS-XU) can delay delamination onset and improve the structural performance of wood single lap joints and whether hybridizing *Pinus pinaster* Aiton and *Quercus alba* substrates enhances stress redistribution under dynamic loading. Three configurations were studied experimentally and numerically under quasi-static (1 mm/min) and impact (3 m/s) loading. The key findings are summarized below.

- Under quasi-static loading, the adhesive-to-wood interface governed the response for all configurations, with all joints failing adhesively (WFP ≈ 0%) and failure loads remaining comparable across reference, toughened and hybrid joints. This confirms that substrate reinforcement strategies offer limited benefit when the adhesive bond line is the critical weak link and that improving quasi-static performance requires targeting the interface rather than the substrate architecture.
- In contrast, under impact loading, failure shifted entirely to wood delamination (WFP ≈ 100%), making substrate reinforcement strategies far more effective. The HMA interlayer successfully delayed delamination onset and increased energy absorption capacity, confirming that thermoplastic film adhesives are well suited to dynamic loading scenarios where viscoelastic stress redistribution and adhesive penetration into the wood cell structure are advantageous.
- The hybrid pine-oak configurations achieved the greatest performance gains under impact, with the P-O-P arrangement reaching an 84% improvement over the pine reference. Finite element analysis confirmed that this enhancement originates from stress-wave redirection toward the stiffer inner plies and a 38% reduction in peak peel stress at the overlap ends, which collectively delay delamination initiation and promote more uniform energy absorption across the substrate. This improvement is attributed to the redistribution of stresses through the substrate, as confirmed by the numerical simulations. The models showed that hybrid configurations reduced peel stresses at the overlap ends and shifted stress-wave propagation toward the inner plies, delaying delamination and allowing more uniform energy absorption.
- Although delamination was not completely prevented, the introduction of hybrid substrates significantly delayed its onset and increased the overall energy absorption capacity of the joints. These findings highlight the potential of combining oak and pine plies, bonded with a thermoplastic interlayer, as a lightweight and effective strategy for improving the dynamic performance of wood adhesive joints.

Future research should focus on optimizing the interface between adhesive and wood, investigating different ply arrangements and tailoring adhesive properties to further suppress delamination, as well as developing a numerical model for the toughened joints. Such developments could extend the applicability of sustainable wood-based bonded structures in demanding structural applications.

## Figures and Tables

**Table 1:** Nominal orthotropic properties of pine wood [21].

**Properties**	**Value**
*E_L_* (GPa)	12.00
*E_R_* (GPa)	1.91
*E_T_* (GPa)	1.01
*ν_LT_*	0.51
*ν_LR_*	0.47
*ν_TR_*	0.31
*G_LR_* (GPa)	1.12
*G_LT_* (GPa)	1.12
*G_TR_* (GPa)	0.29
*σ_L_* (MPa)	97.50
*σ_R_* (MPa)	7.90
*σ_T_* (MPa)	4.20

**Table 2:** Nominal orthotropic properties of oak wood [21,22].

**Properties**	**Value**
*E_L_* (GPa)	12.30
*E_R_* (GPa)	2.05
*E_T_* (GPa)	0.89
*ν_LT_*	0.43
*ν_LR_*	0.37
*ν_TR_*	0.30
*G_LR_* (GPa)	1.20
*G_LT_* (GPa)	1.20
*G_TR_* (GPa)	0.80
*σ_L_* (MPa)	105.00
*σ_R_* (MPa)	13.80
*σ_T_* (MPa)	5.50

**Table 3:** Elastic properties of the hot-melt adhesive film.

**Properties**	**Value**
*E* (MPa)	230.18
σ_y_ (MPa)	5.49
σ_f_ (MPa)	10.86
*ν*	0.47
*ε*	10.66
*G* (MPa)	76.67
τ (MPa)	5.09
γ	3.54

## Data Availability

The original contributions presented in this study are included in the article. Further inquiries can be directed to the corresponding author.
